# Navigating the Unexpected: Right Aortic Arch Unveiled During Left Heart Catheterization

**DOI:** 10.7759/cureus.57802

**Published:** 2024-04-08

**Authors:** Firas Anaya, Anas Alameh, Anibelky Almanzar, Christopher Cunningham, Aisha Siraj

**Affiliations:** 1 Department of Medicine, MetroHealth Medical Center, Cleveland, USA; 2 Department of Cardiovascular Medicine, MetroHealth Medical Center, Cleveland, USA

**Keywords:** interventional cardiology, congenital aortic arch anomaly, left heart cath, mirror-image branching, right aortic arch

## Abstract

A right aortic arch (RAA) is an extremely rare congenital anomaly with seven identified variants. While most variants are asymptomatic, those with a vascular ring can be associated with severe symptoms. We present an incidental RAA finding during left heart catheterization (LHC) in a 68-year-old female with multiple morbidities presented with worsening angina. Her echocardiogram was significant for inferolateral wall akinesia, prompting LHC. The procedure was challenging with an unexpected course of the guide wire distally behind the cardiac shadow. Pressure tracing confirmed arterial access and contrast injection revealed RAA. A subsequent aortic computed tomography angiography (CTA) confirmed RAA with mirror-image branching. Abnormal origin and angle of aortic arch branches pose challenges in choosing the proper access. We used the right radial artery approach, but the left radial approach may be superior in providing a more proximal access and avoiding the abnormal origin of the right subclavian artery (RSA). Choosing the appropriate angiographic view is also of utmost importance, and the right anterior oblique view provided better visualization in our case. Aortic arch anomalies are confirmed by a CTA or magnetic resonance angiography (MRA) of the aorta. This case underscores the importance of identifying the aortic arch anomalies and the imposed challenges during the LHC.

## Introduction

A right aortic arch (RAA) is an exceedingly rare congenital vascular anomaly with an incidence rate of 0.1% [1,2]. RAA manifests when the aortic arch develops to the right of the trachea and esophagus instead of the typical left-sided positioning. Seven distinct variants of an RAA have been reported in the literature [1]. An RAA with mirror-image branching is the second most common variant, coming after the RAA with an aberrant left subclavian artery (LSA) [2].

RAA variants can be variably associated with another cardiac anomalies. However, unlike our presented case, an RAA with mirror-image branching is associated with other cardiac defects in 98% of the cases, such as tetralogy of Fallot, pulmonary atresia, or truncus arteriosus [1]. RAA anomalies are usually asymptomatic unless associated with vascular rings or intracardiac anomalies [1-3].

In an RAA with mirror-image branching, the LSA and left common carotid (LCC) arteries originate from the left innominate artery (LIA), which originates most proximally from the arch. The LIA is followed by the right common carotid, and the most distal arch branch will be the right subclavian artery (RSA) [4]. Understanding the intricate anatomy of RAA branches is of paramount importance for interventionalists. Expected procedural challenges during left heart catheterization (LHC) in patients with an RAA include an extended procedural duration and potential difficulties in achieving successful LHC [5]. In this case report, we present an incidental finding of an RAA during the LHC procedure and discuss the observed findings and the imposed challenges during LHC.

## Case presentation

We present a 68-year-old female patient with a past medical history notable for heart failure with preserved ejection fraction, essential hypertension, type II diabetes mellitus, and obstructive sleep apnea. The patient's primary complaint was exertional left-sided chest tightness and shortness of breath, which was relieved with rest. Physical examination revealed trace bilateral pitting edema in the lower extremities but was normal otherwise. Laboratory test results were largely within normal limits, except for a high-sensitivity troponin level of 26, which subsequently trended down. The electrocardiogram displayed sinus rhythm, along with first-degree atrioventricular block, right bundle branch block, and a heart rate of 88 beats per minute.

**Figure 1:**
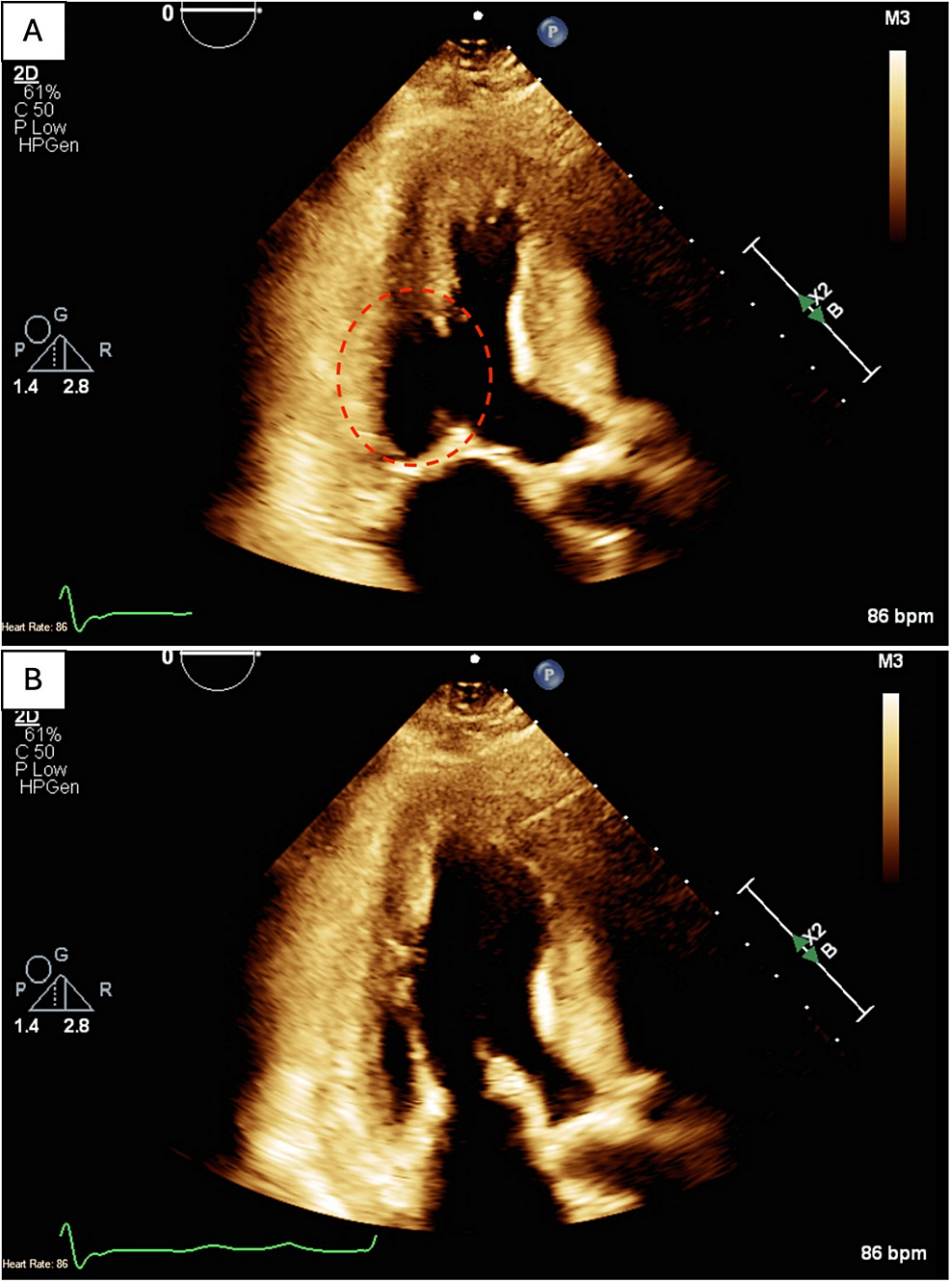
Two-chamber view echocardiogram showing the left ventricle and the left atrium. A. Displays the left ventricle during systole with akinesia in the inferolateral segment (red-dashed circle). B. Displays the left ventricle expanding during diastole.

**Figure 2:**
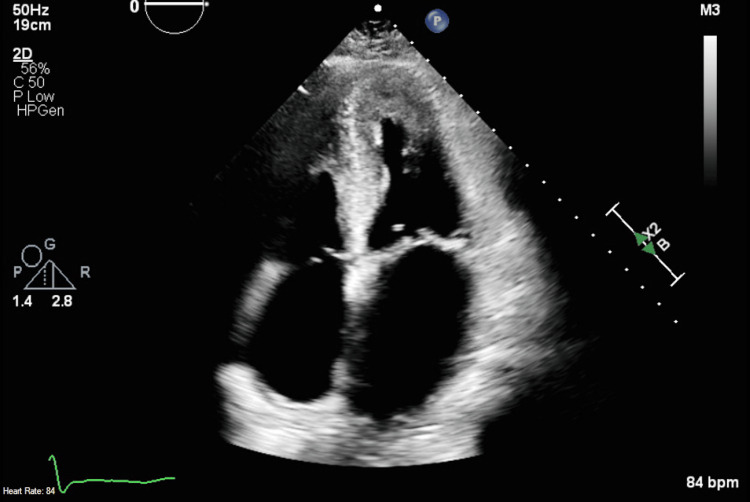
Apical four-chamber view echocardiogram showing left atrial dilation and left ventricular hypertrophy.

An echocardiogram showed focally abnormal left ventricular (LV) systolic function with akinesia in the inferolateral wall (Figure 1), LV ejection fraction of 55%, concentric left ventricular hypertrophy, and dilated left atrium (Figure 2).

**Figure 3:**
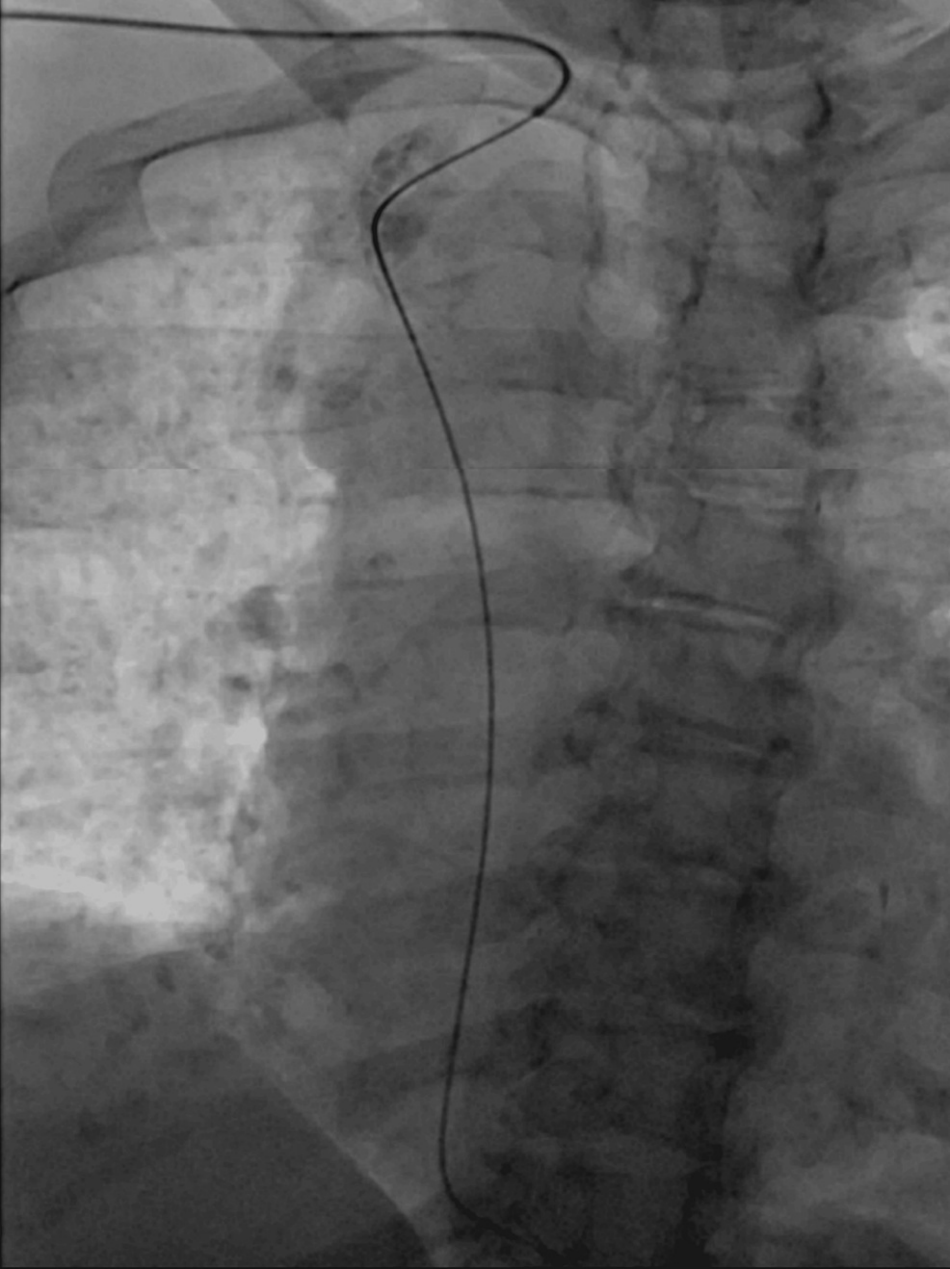
Left anterior oblique view during left heart catheterization showing the guide wire diving downward in the right side of the chest suggesting the right-sided orientation of the aortic arch.

**Figure 4:**
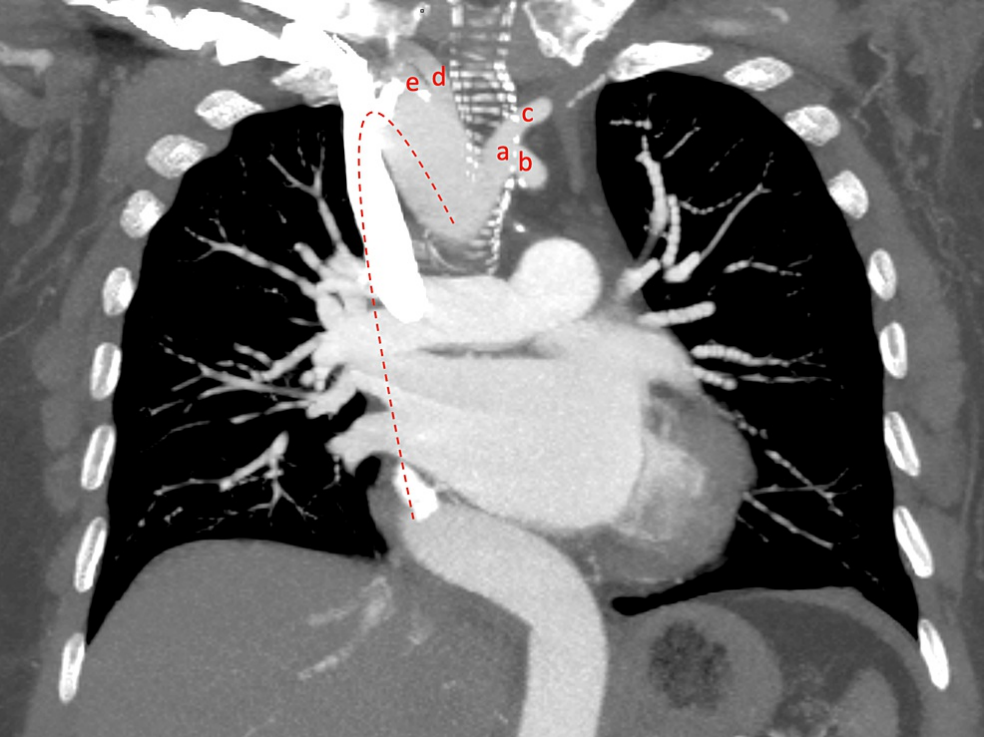
Computed tomography angiography showing the right-sided orientation of the aortic arch (red dashed line) with mirror-image branching. Aortic arch branches from proximal to distal: a: left innominate artery, b: left subclavian artery, c: left common carotid artery, d: right common carotid artery, e: right subclavian artery.

Given the patient's clinical history and abnormal echocardiographic findings, a decision was made to proceed with an LHC to assess for coronary artery disease. During the LHC, an unexpected deviation of the J-wire across the LV shadow in a right paraspinal position prompted concerns about abnormal vascular anatomy. Arterial pressure tracing confirmed the catheter's arterial position and contrast revealed the presence of an RAA (Figure 3). Subsequent attempts to access the ascending aorta while using the left anterior oblique view proved unsuccessful. A right anterior oblique view was then employed, which provided enhanced visualization of the RAA course, leading to a successful LHC. No significant coronary artery disease was revealed during the procedure. Aortic computed tomography angiography (CTA) was done later after discharge and confirmed the presence of an RAA with mirror-image branching (Figure 4). 

## Discussion

Selecting the most appropriate vascular access route is the initial challenge encountered when performing LHC in patients with abnormal aortic arch anatomy. Generally, the transradial approach is preferred over the femoral artery approach in LHC due to its association with fewer bleeding complications [6]. Specifically, the right radial artery is favored over the left due to its ergonomic suitability for right-handed operators [7,8].

However, the inherent complexity and unpredictable nature of the RAA and its variants compound the challenge of achieving successful LHC. Each variant has its own challenges. Given the reverse branching in an RAA with mirror-image branching, the femoral artery and left radial artery approaches may be superior, providing a more proximal access and avoiding the abnormal direct and acute-angle origin of the RSA, as the guide wire may selectively dive into the descending aorta in the RSA approach [9,10].

In our presented case, the right radial approach was utilized. The unexpected downward deviation across the LV shadow indicated that the guide wire had exited the origin of the RSA, following a path toward the descending aorta rather than proceeding as expected toward the ascending aorta, requiring multiple manipulation attempts. This deviation was primarily attributed a the abnormal origin and angulation of the RSA. Another challenge encountered in achieving a successful LHC in RAA patients is obtaining an adequate radiographic view. In our case, the left anterior oblique was inadequate, whereas the right anterior oblique view provided a better visualization and proved to be more helpful in accessing the ascending aorta. Both challenges contributed to prolonged procedural time; however, no periprocedural complications were encountered.

Ultimately, when such anomalies are encountered during the procedure, confirmation through CTA or MRI angiography is necessary to gain a clear understanding of the arch anatomy. If an RAA is suspected based on prior chest X-rays or CT scans, it is necessary to perform a dedicated CTA to identify the anomaly before proceeding with the LHC. Multidetector CT scanning has recently emerged as the gold standard for diagnosing RAA, with MRI angiography representing a viable alternative [3,11]. In our case, the LHC did not reveal ischemic or stenotic lesions in any of the coronary arteries, and no interventions were done during the procedure. The CTA was ordered as an outpatient, which confirmed the presence of RAA with mirror-image branching as illustrated above in Figure 4. The patient received intravenous furosemide inpatient until euvolemia was achieved. After discharge, she was seen by the cardiology team and reported being symptom-free. The outpatient cardiology team further optimized the guideline-directed medical therapy for the cardiomyopathy, without plans for additional workup or future interventions.

## Conclusions

This case underscores the importance of considering the variations in vascular anatomy, such as an RAA, when encountering unexpected challenges during the LHC. This includes choosing the appropriate vascular access and obtaining the optimal angiographic view. Furthermore, the case highlights the significance of the multimodal diagnostic approach to ensure thorough evaluation, identify the high-risk variants, and facilitate successful intervention in a timely manner when warranted.
